# Hpz1 Modulates the G1-S Transition in Fission Yeast

**DOI:** 10.1371/journal.pone.0044539

**Published:** 2012-09-06

**Authors:** Cathrine A. Bøe, Jon Halvor J. Knutsen, Erik Boye, Beáta Grallert

**Affiliations:** 1 Department of Cell Biology, Institute for Cancer Research, Oslo, Norway; 2 Institute for Molecular Biosciences, University of Oslo, Norway; Newcastle University, United Kingdom

## Abstract

Here we characterize a novel protein in *S. pombe*. It has a high degree of homology with the Zn-finger domain of the human Poly(ADP-ribose) polymerase (PARP). Surprisingly, the gene for this protein is, in many fungi, fused with and in the same reading frame as that encoding Rad3, the homologue of the human ATR checkpoint protein. We name the protein Hpz1 (Homologue of PARP-type Zn-finger). Hpz1 does not possess PARP activity, but is important for resistance to ultraviolet light in the G1 phase and to treatment with hydroxyurea, a drug that arrests DNA replication forks in the S phase. However, we find no evidence of a checkpoint function of Hpz1. Furthermore, absence of Hpz1 results in an advancement of S-phase entry after a G1 arrest as well as earlier recovery from a hydroxyurea block. The *hpz1* gene is expressed mainly in the G1 phase and Hpz1 is localized to the nucleus. We conclude that Hpz1 regulates the initiation of the S phase and may cooperate with Rad3 in this function.

## Introduction

Cell growth and proliferation involve a series of distinct reaction pathways that are linked together in what is termed the cell cycle [1]–[3]. Preparation for another round in the cell cycle is made already as the cells exit mitosis, when the Origin Recognition Complex (ORC) is bound at the future origins of DNA replication, to be activated in the following S phase. In late mitosis or G1 phase the replicative helicase, the MCM hexamer, is loaded onto the replication origins marked by ORCs. This event is dependent upon a transcripton factor that activates genes encoding the proteins responsible for MCM loading. In human cells the loading is dependent upon the CDC6 and CDT1 proteins and homologous proteins have similar activities in all other eukaryotes. Thereafter, a series of events, including the activation of an S-phase cyclin-dependent kinase (CDK), leads to initiation of DNA replication at a subset of the replication origins [4]–[6]. Some origins are initiated early in S phase, others at a later stage. After successful completion of S phase the cell prepares for mitosis and CDK activity is required also for the G2-M transition [7]–[9]. In mitosis the chromosomes are segregated, the nucleus divides, and the cell can prepare for division.

Regulation of the cell cycle is performed by a number of feedback and feed-forward mechanisms and in addition by external checkpoint mechanisms that arrest the cell cycle if the DNA is damaged or if one phase of the cell cycle has not been properly finished [10]. The central checkpoint proteins in human cells are the ataxia telangiectasia mutated (ATM) and the ATM and RAD3-related (ATR) proteins. Both ATR and ATM are large phosphoinositide 3-kinase-related protein kinases (PIKKs) with multiple substrates.

ATR associates with its obligate partner ATRIP to perform its function. The ATR protein, as well as its homologues in other eukaryotes, contains a C-terminal kinase domain and an N-terminal ATRIP-binding domain, separated by a large α-helical HEAT domain. A similar structure is found for the ATR homologue in fission yeast, Rad3, whose binding partner is Rad26. There are undoubtedly a large number of proteins that the heterodimer Rad3/Rad26 interacts with, but few of them are known.

Human cells are not viable without ATR, but the essential function has not been identified. ATR is involved in the activation of chromosomal replication origins within S phase as well as in the stabilization of stalled replication forks [11]–[13], but the detailed molecular functions are still poorly understood. ATR phosphorylates a subunit of the replicative helicase, MCM2 [14], [15], in a reaction that may regulate S-phase progression [16]. ATR is activated by DNA damage and in particular by single-stranded DNA generated by repair processes and bound by Replication Protein A [17], but the mechanism of activation is not well characterized. Furthermore, ATR phosphorylates proteins involved in recombination [18]–[21] and nucleotide excision repair [22]. The intracellular activity of PIKK kinases is known to be regulated, at least in part, by their localization [23] and this is likely to be true also for ATR.

In this work we describe a fission yeast protein whose homologue in many fungi is encoded within the same open reading frame as the Rad3 homologue, suggesting that the two proteins are acting together also when they are encoded separately. This protein shows a high degree of homology with the Zn- finger domain of the human Poly(ADP-ribose) polymerase (PARP). We present evidence that the gene is involved in DNA replication control and may interact with Rad3. In particular, absence of the protein conveys some of the same phenotypes that are found for the *rad3* deletion mutant, arguing that the two proteins are acting in some common reaction pathway(s).

## Results

### Identification of a Potential Functional Partner of Rad3

**Figure 1:**
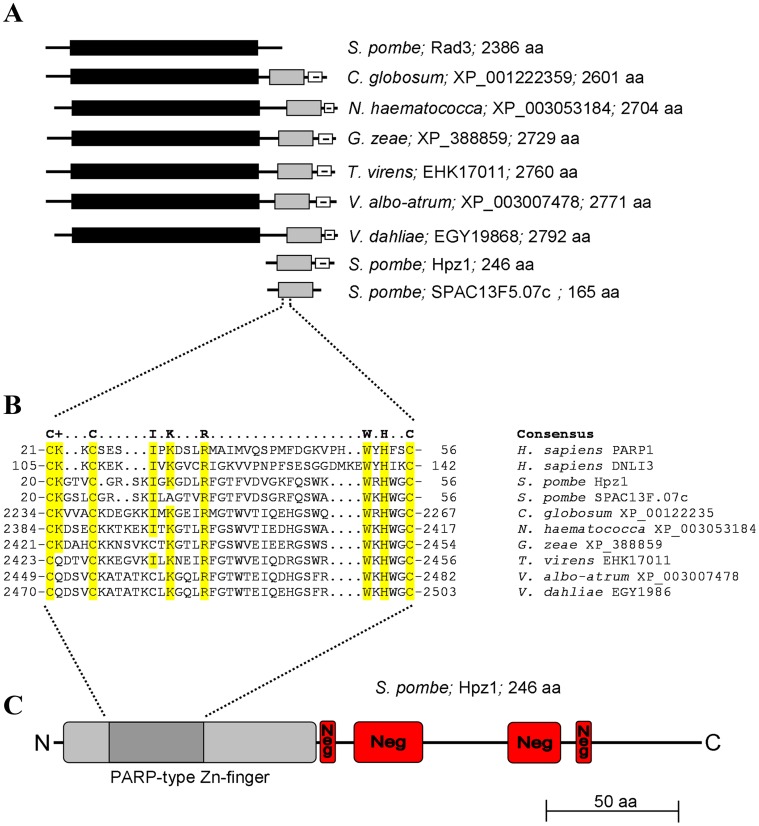
Homology and composition of Hpz1. A. Schematic presentation of Rad3 (black bar) from *S. pombe*, six Rad3-like proteins in different fungi with a PARP-type Zn-finger domain (gray bar) and a negatively charged C-terminal domain (white bar with minus sign), and the two homologues of the PARP-type Zn-finger found in *S. pombe*, Hpz1 and SPAC13F5.07c (not drawn to scale). B. Multiple-sequence alignment showing the consensus sequence of the PARP-type Zn-finger domain (IPR001510, http://www.ebi.ac.uk/interpro/) and aligned sequences below. Conserved residues are highlighted in yellow. The numbers in front of and after the sequence indicate the residue numbers. C. Schematic representation of the Hpz1 protein in *S. pombe* (drawn to scale). Indicated are the PARP-type Zn-finger domain (light gray), the Zn-finger signature sequence used in the multiple-sequence alignment (dark gray) and four regions that show bias towards the negatively charged amino acids glutamate and aspartate (red).

In fission yeast Rad3 is a major regulator of the response to DNA damage and stalled replication forks. We compared the homologues of Rad3 in a wide range of organisms and found that in several fungi the protein is extended at the C-terminus with an additional motif (Fig. 1 A), that shows extensive homology to the Poly(ADP-ribose) polymerase (PARP)-type Zn-finger (IPR001510) (Fig. 1 B). The C-terminal extension also contains a region enriched in negatively charged residues. The fission yeast genome contains two genes encoding proteins with extensive homology to the PARP-type Zn-finger motif, SPBC2A9.07c and SPAC13F5.07c (Fig. 1 A). Of the two, only SPBC2A9.07c contains the negatively charged clusters conserved in the fungal Rad3 homologues and is therefore the homologue investigated further in this work. We named SPBC2A9.07c Hpz1 for Homologue of PARP-type Zn-finger. No obvious Hpz1 homologue can be identified in *Saccharomyces cerevisiae.* The highest degree of similarity to Hpz1 in the current genome databases was found in the C-terminal end of the Rad3-homologue XP_00122235 in *C. globosum*. The PARP-type Zn-finger motif shows a higher degree of conservation between the fungal homologues and Hpz1 than between Hpz1 and the human PARP1 or DNA ligase 3 (Fig. 1 B). However, this motif is found in several eukaryotes and even in bacteria. For example, there are 15 proteins with this motif in mouse and 13 proteins in the human genome. Of these, there are several small proteins with a PARP-type Zn-finger motif but no other obvious domains, including the negatively charged C-terminal domain. It is unclear whether these proteins share functions with each other and whether they can be considered are functional homologues of Hpz1.

Hpz1 is predicted to contain 246 amino acid residues with a molecular weight of 28.1 kDa. The protein contains a PARP-type Zn-finger domain on the N-terminus and a region enriched in negatively charged amino acid residues on the C-terminus (Fig. 1 A and C). We considered the intriguing possibility that Hpz1 might have PARP activity. However, the homology of Hpz1 to established PARP genes is limited to the Zn-finger domain. In eukaryotes PARPs belong to a protein family catalyzing poly(ADP-ribosyl)ation of DNA-binding proteins. The active site of PARPs is located within a highly conserved 50 amino acid sequence called “the PARP signature” [24], [25]. There is no obvious PARP signature in the protein sequence of Hpz1. Consistently, we could not detect poly(ADP-ribosyl)ated proteins in cell extracts from *S. pombe* (data not shown). These results are consistent with previous findings that fission yeast does not contain a PARP homologue [26].

The fusion of Rad3 to Hpz1 homologues in several fungi indicates that the two proteins share function(s) or participate in the same biological process(es). Therefore we decided to explore whether Hpz1 has functions related to those of Rad3.

### *hpz1*Δ is Sensitive to Ultraviolet Light in G1 Phase and to HU Treatment

**Figure 2:**
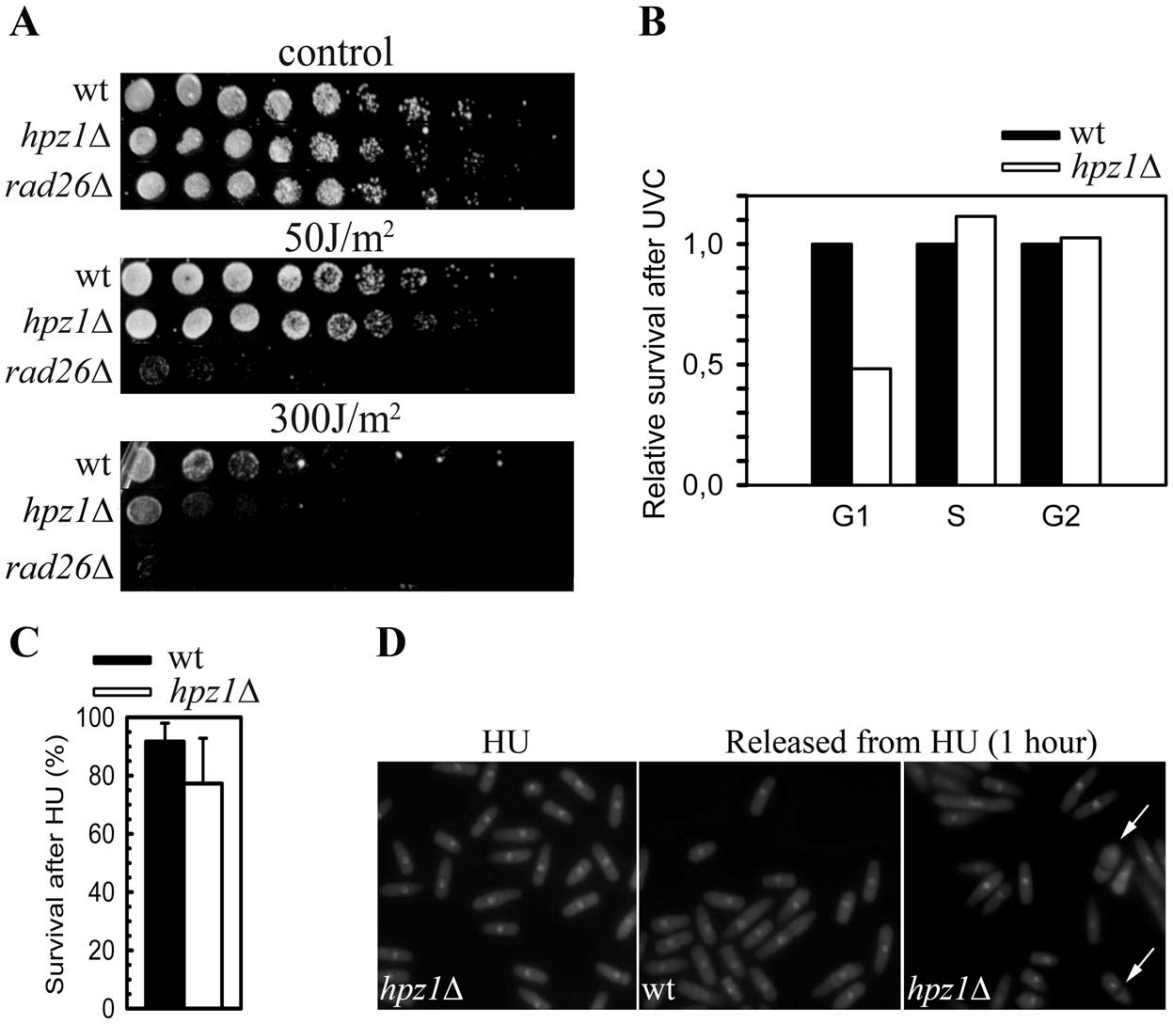
UVC- and HU-sensitivity. A. Spot test for UVC sensitivity of wild-type (wt), *hpz1*Δ and *rad26*Δ cells. Upper: unirradiated cells, center: 50J/m^2^, lower: 300J/m^2^. B: Survival after UVC irradiation of wild-type or *hpz1*Δ cells in G1, S or G2 phase. The survival of wild-type cells was normalized to 1 in each cell cycle phase (data without normalization are shown in Fig. S1). C. Survival of wild-type or *hpz1*Δ cells after HU-treatment. D. Microscopy images of *hpz1*Δ cells in HU (left) and 1 hour after release from HU (center) and wild-type cells released from HU (right). The DNA was stained with 4′,6-diamidino-2-phenylindole **(**DAPI). The arrows point to cut cells.

One known function of Rad3 is to induce an appropriate response to DNA damage or stalled replication forks, and *rad3*Δ cells are extremely sensitive to DNA-damaging agents. We found that the *hpz1*Δ mutant was slightly more sensitive to ultraviolet light (UVC) than wild-type cells (Fig. 2 A), but not as sensitive as a checkpoint deficient mutant (*rad26*Δ). We considered the possibility that Hpz1 is only required in a small fraction of the cells in an asynchronous population. The UVC sensitivity in different cell-cycle phases was determined in wild-type and *hpz1*Δ cells synchronized in G1 phase, using a *cdc10* block-and-release (see M&M) followed by UVC-irradiation in G1, S or G2 phase. Wild-type cells were most resistant to UVC in G2 and least in S phase (Fig. S1). The survival of *hpz1*Δ cells irradiated in G1 phase was reduced by 50% compared to a wild-type strain, but no differences were found in the other cell-cycle phases (Fig. 2 B). These results indicate an important function for Hpz1 after UVC irradiation specifically in G1 phase.

The sensitivity to ionizing radiation of *hpz1*Δ mutant cells was no different from that of wild-type cells (Fig. S2) suggesting that Hpz1 does not play an important role in double-strand break repair.

Rad3 is activated when replication forks stall and this leads to checkpoint activation (see Introduction) and cell cycle arrest. Hydroxyurea (HU) inhibits the ribonucleotide reductase leading to depletion of the nucleotide pools and to the stalling of replication forks [27] and to checkpoint induction. HU-treated *rad3*Δ cells do not arrest in the intra-S checkpoint, but rather continue into mitosis and divide with the DNA unevenly distributed between the daughter cells [28], displaying the so-called “cut” phenotype [29], [30], which results in poor cell survival. To investigate the requirement for Hpz1 when replication forks stall we determined the tolerance of *hpz1*Δ to HU. The survival of *hpz1*Δ after 4 hours in HU (15 mM) was 10% lower than for wild-type cells (Fig. 2 C), but the *hpz1*Δ cells did not appear cut (Fig. 2 D left) and they arrested with 1C DNA (early S phase) as judged by flow cytometry (data not shown). However, 1 hour after release from HU ∼7% of *hpz1*Δ cells displayed the cut phenotype (Fig. 2 D). It is not unlikely that the cutting corresponds to the 10% reduction in survival.

### Hpz1 and Rad3 Might Interact

The UV and HU sensitivity of the *hpz1*Δ mutant (above) indicates a role for Hpz1 under these conditions. We therefore chose UVC- and HU-treatments to investigate the interaction between Hpz1 and Rad3. Cells carrying Hpz1-HA and Rad3-myc were synchronized in G1 phase by a *cdc10* block, released into the cell cycle and either UVC-irradiated in G1 or S phase or released into an HU–induced S-phase arrest. Hpz1-HA was immunoprecipitated from the extracts of these cells and the immunoprecipitate was analyzed for the presence of Rad3-myc. Co-immunoprecipitated Rad3-myc could be detected in extracts from untreated G1 cells and HU-treated cells, but not from S-phase cells (Fig. S3). However, this interaction was only detected in two experiments and cannot be considered conclusive. Nonetheless, the data suggest that an interaction between Hpz1 and Rad3 might indeed exist, but that it is indirect or transient.

### Initiation of DNA Replication is Advanced in *hpz1*Δ Mutant Cells

**Figure 3:**
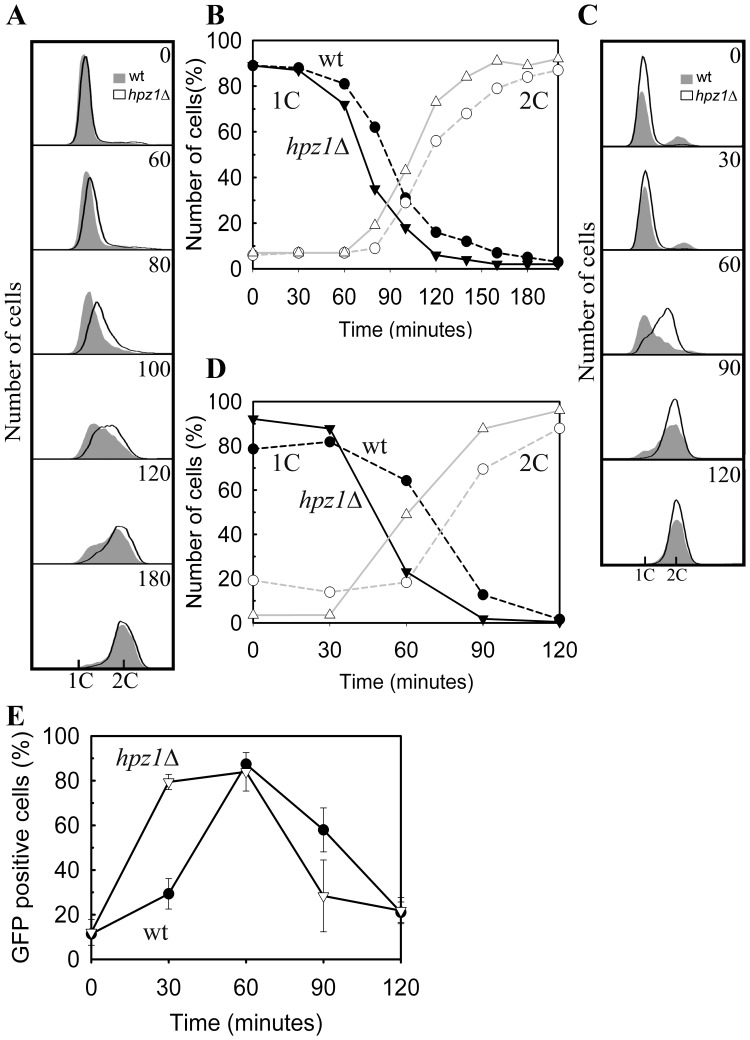
Progression of DNA replication. Analysis of the increase in DNA content in individual wild-type and *hpz1*Δ cells after two different methods for synchronization and release. A. DNA histograms of cells blocked in early S phase by HU-treatment for 4 hours, then washed and released into the cell cycle for the times indicated. B. Quantification of the cells (in A) with a 1C DNA or 2C DNA content after HU-treatment and release into the cell cycle for the times indicated. C. DNA histograms of *cdc10* cells that were synchronized in G1 phase, released into the cell cycle and incubated for the time indicated. D. Quantification of the cells (in panel C) with a 1C DNA or 2C DNA content after a *cdc10* block and release into the cell cycle and incubated for the times indicated. E. PreRC formation in wild-type and *hpz1*Δ cells as a function of time after release from a *cdc10* block.

The HU sensitivity assay indicated an abnormal response of *hpz1*Δ to stalled replication forks, but not a checkpoint defect similar to that of *rad3*Δ cells. To further explore this response the cellular DNA content of wild-type and *hpz1*Δ cells was monitored after they were released from an HU block. In several repeated experiments the *hpz1*Δ mutant cells invariably increased their DNA content earlier than wild-type cells did (Fig. 3 A). The quantification of cells with a 1C (early S phase) or 2C DNA content (G2) from these experiments showed that the time lag between wild-type and *hpz1*Δ is about 15 min throughout S-phase (Fig. 3 B).

The above results suggest that in the *hpz1*Δ mutant initiation or restart of replication forks are advanced. To determine whether the earlier increase in DNA content also occurs when the cells are synchronized before S phase, we arrested cells in G1 phase in a *cdc10* block, released them from the block and followed their progression into and through S-phase (Fig. 3 C and D). Surprisingly, *hpz1*Δ cells seemed to increase their DNA content earlier than wild-type cells did.

To exclude the possibility that *hpz1*Δ cells normally progress faster through S phase and therefore spend shorter time in S phase we analyzed asynchronous populations of cells by flow cytometry and measured the numbers of cells in the different cell-cycle phases [31]. The results showed no differences in the percentage of wild-type versus *hpz1*Δ cells in S phase, arguing that the time spent in S phase was the same (Fig. S4).

We also measured the timing of MCM loading in G1 phase in the two strains after a *cdc10* block-and-release. The MCM complex is loaded onto future replication origins to form the Pre-replicative complex (PreRC) and this event can be followed in a microscope when employing a fluorescently tagged MCM [32]. We found that maximal loading of MCMs occurred 60 min after the release of wild-type cells (Fig. 3E), in agreement with earlier observations [33]. However, in the *hpz1*Δ cells the maximum consistently occurred about 15 minutes earlier, suggesting that Hpz1 is negatively modulating an event at or before PreRC formation. It should be noted that this phenotype is different from that observed above for cells synchronized inside S phase, and this will be discussed below.

### Hpz1 Localizes to the Nucleus and is Expressed in a Cell-cycle-dependent Manner

**Figure 4:**
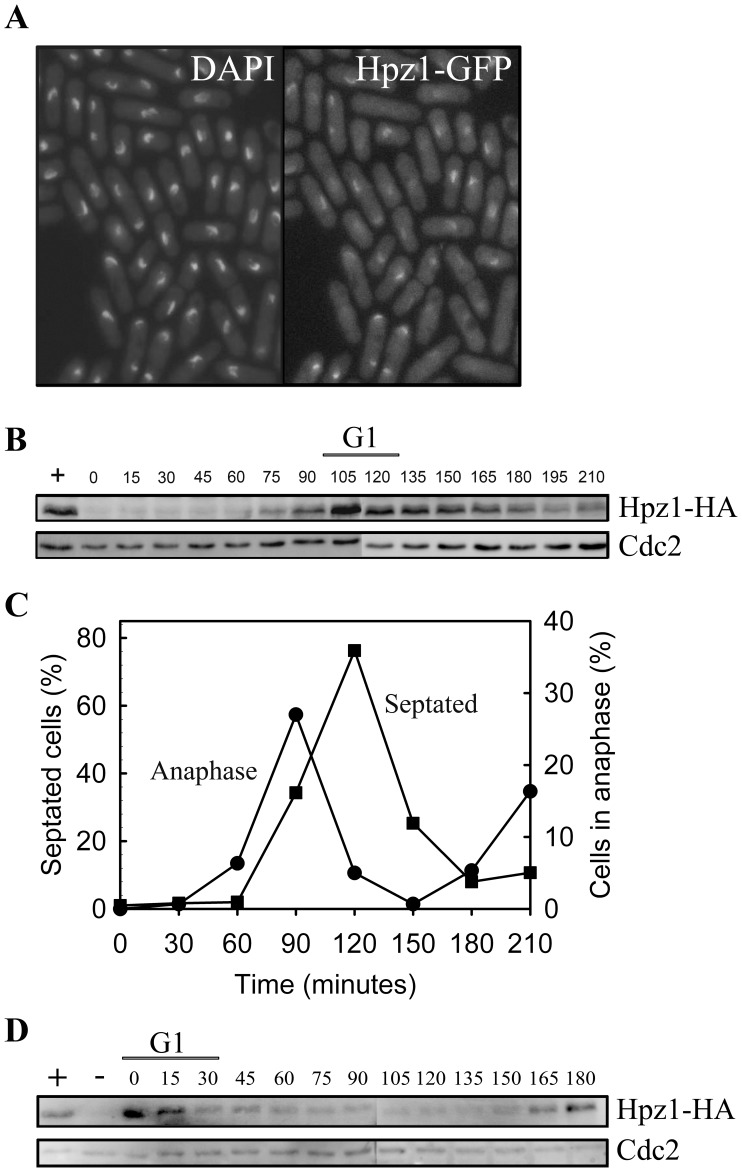
Cell-cycle regulation and localization of Hpz1. Fluorescence microscopy pictures showing all nuclei (DAPI-staining, left) and Hpz1-GFP localization (right) in the same cells. B. Immunoblot showing the expression of Hpz1-HA in total cell extracts taken at the indicated time points after a *cdc25* block-and-release. C. The percentage of cells in anaphase (DAPI-stained cells with 2 nuclei) or septated cells (stained with aniline blue) from the experiment shown in panel B. D. Immunoblot showing the presence of Hpz1-HA in total cell extracts taken at the indicated time points after a *cdc10* block-and-release.

The Zn-finger domain in Hpz1 indicates that it is capable of DNA binding, and hence suggests a nuclear localization. In a global ORFeome analysis, over-expressed Hpz1 was found to localize to the mitochondria and some nuclear signal was also observed [34]. We have fused a GFP-tag to the C-terminus of Hpz1 and the fusion protein was expressed from its endogenous promoter. GFP localization was determined by fluorescence microscopy of exponentially growing cells. We observed a strong and clear nuclear signal in a significant fraction of the cells and no signal in the other cells (Fig. 4 A). Furthermore, the nuclear signal was apparently dependent upon the cell-cycle stage, since Hpz1-GFP was mainly detectable in cells with two nuclei (M or G1 phase) and in some of the smallest cells (late S – early G2).

To explore the suggested cell-cycle regulated expression further the presence of Hpz1-HA was investigated by immunoblotting of the total extracts of cells synchronized in G2/M by a *cdc25* block-and-release experiment (Fig. 4 B). The frequency of cells in anaphase and the septation index were determined at different times after release into the cell cycle (Fig. 4 C) as a measure of synchronous progression through the cell cycle. The cellular level of Hpz1 was found to increase in late anaphase, was maximal in G1 phase and declined in S phase (Fig. 4 B and C).

Proteins specifically expressed in G1 are often regulated by the Cdc10 transcription factor [35]. To determine whether the cell-cycle-regulated expression of Hpz1 depends on Cdc10, we monitored the expression of an Hpz1-HA fusion protein after a *cdc10* block-and-release experiment (Fig. 4 D). Hpz1 was present at the time of G1 arrest, but disappeared shortly after release from the *cdc10* block, arguing that *hpz1* is not a Cdc10 target. We conclude that the expression of Hpz1 is limited to the M/G1 phase. The PCB (Pombe Cell-cycle Box)-binding factor (PBF) is a transcription factor responsible for M/G1-specific transcription of its target genes [36]. A search for PCB-motifs, the known binding site of PBF [36], revealed two PCB-motifs upstream of *hpz1* (Fig. S5), suggesting that it is a target of PBF.

## Discussion

We have identified and characterized Hpz1, a novel putative partner for Rad3 in fission yeast. This partnership may give important insights into the functions of Rad3 and of its homologues in the ATR family of proteins since these proteins perform important, and sometimes essential, functions in cell-cycle regulation and in maintenance of the genome.

### Functional Clues from Protein Sequence Information

Hpz1 contains a PARP-type Zn-finger domain, but lacks other features necessary for PARP function. There is no evidence for the existence of poly (ADP-ribosyl)ated proteins in cell extracts from fission yeast ([26] and this work). PARPs play an important role in human cells by detecting and binding to single-stranded DNA breaks and thereby to signal to repair enzymes [37]. We show here that the *hpz1* deletion mutant is more sensitive to UVC than wild-type cells are. However, we reason that such a minor increase in sensitivity is unlikely to be caused by the absence of a major DNA repair function. Together these results point to a different function of Hpz1 than poly (ADP-ribosyl)ation and DNA repair. The PARP-type Zn-finger is a domain found in several human proteins. Some of them have a known function, like PARP-1 and DNA ligase 3, whereas others are yet uncharacterized, but resemble Hpz1 in size and in that they only contain one PARP-type Zn-finger domain. Thus, the available sequence information suggests that Hpz1-like proteins might also be conserved in higher eukaryotes.

### Hpz1 and Rad3 Might Act Together in G1 Phase

The fact that homologues of the two very different proteins Hpz1 and Rad3 are fused in several fungi suggests a shared function of the separate proteins in other organisms. We show here that *hpz1*Δ does not share the checkpoint defects known for *rad3*Δ cells, so Hpz1 is not required for the checkpoint functions of Rad3. However, Hpz1 is exerting its function in G1, where no function of Rad3 is yet described. Several Mcms have been shown to be substrates of Rad3 or of Rad3 homologues in other species. But these modifications either occur in S phase [38] or have been shown to be important for the intra-S checkpoint [14], pointing to a role for Rad3 in the cell-cycle progression after PreRC loading. We have been able to produce weak evidence that the two proteins are interacting and specifically in G1 phase, but the interaction is probably transient and not very strong. These observations point to a function also for Rad3 in G1 phase. Indeed, we have shown that the early-replication phenotype of the *hpz1*Δ mutant can also be observed in *rad3*Δ cells after a *cdc10* block-and-release (manuscript in preparation). These observations argue that the two proteins might share at least one function in the regulation of G1-S progression.

### Hpz1 Regulates the Start of DNA Replication

Our results from two distinct types of experiments clearly show that Hpz1 has a function in the start of DNA replication. First, when the cells are arrested in early S phase by HU and the drug is washed out, the *hpz1* deletion mutant resumes DNA replication earlier than wild-type cells do. This restart may involve already assembled replication forks that have been stalled by a lack of deoxyribonucleotides during HU treatment. Alternatively, the premature firing of late origins might be promoted in *hpz1*Δ mutant cells after release from the HU block. Second, cells arrested in G1 phase by a *cdc10* block and released into the cell cycle also start DNA replication earlier in the absence of Hpz1. One possible explanation for both of these sets of results is that there are more replication forks active in the absence of Hpz1, yielding faster chromosome replication rates and a shorter S phase. This explanation was ruled out by separate experiments (Fig. 3 and Fig. S4). Furthermore, in the *cdc10*-experiments we could show that an event before initiation of DNA replication, namely the formation of PreRC formation, was delayed by the presence of Hpz1. We conclude that Hpz1 has a negative regulatory role for the start-up of DNA replication, both at the initiation stage in G1 and at restart from an HU-block. The premature restart of DNA replication of the *hpz1*Δ mutant may be the reason for a higher rate of mitotic cells after release from the HU block and the increased sensitivity to HU.

The simplest explanation for these findings would be that the two phenotypes of the *hpz1*Δ mutant stem from the same function of the Hpz1 protein. Expression of Hpz1 appears to be initiated in mitosis, possibly regulated by PCB boxes, and the maximal amount of Hpz1 is found in G1 phase. These results are in agreement with an earlier genome-wide analysis of the cell-cycle dependence of the mRNA levels of numerous genes in *S. pombe* [39] and strongly argues for a function of Hpz1 in a cell-cycle-related process in G1 phase. This timing of Hpz1 expression is consistent with a function in the early phases of DNA replication, since the formation of the PreRCs at the chromosomal origins starts in late M with origin binding [40] and ends in G1 with the loading of the MCM complex [32]. The timing and extent of origin binding, PreRC assembly and origin firing are closely connected [40]. We show here that the PreRCs were assembled earlier when Hpz1 was absent and, furthermore, that DNA replication restart occurred earlier without Hpz1. In the latter case the early replication origins were already initiated, allowing the MCMs to travel some distance before the forks were arrested. This may suggest a function of Hpz1 after the initiation step. However, up to one-third of the origins have not fired in an HU block [41], [42] and a possible negative effect of Hpz1 on the firing of late replication origins could affect the kinetics of DNA replication restart. We speculate that the reason for the early resumption of DNA replication in HU-treated *hpz1*Δ mutants is the same as for *cdc10*-synchronized cells: early initiation of replication forks. Alternatively, it is possible that some quality of the MCM complex loaded in G1 phase is different in the presence versus absence of Hpz1, a difference that can affect both the initiation kinetics and the properties of the replication forks.

We conclude that Hpz1 is a novel modulator of the G1-S transition by negatively regulating the initiation of DNA replication.

## Materials and Methods

### Bioinformatical Methods

The protein seuquence of Rad3 from *S. pombe* was used as a query in a standard protein BLAST against the non-redundant protein sequences from fungi (taxid:4751). Results were analyzed using MyHits Motif scan (http://myhits.isb-sib.ch/). Multiple- sequence alignments were performed using ClustalW [43] with default options. The bias towards negatively charged amino acids was determined using ProBias [44], [45].

### Yeast Strains, Cell Handling, Staining and Strain Construction

All strains used in this study (Table S1) were derivatives of *Schisosaccharomyces pombe* L972 h-. Media and conditions were as described previously [46]. The cells were grown exponentially in Edinburgh minimal medium to a density of 2–4×10^6^/ml (OD_595_ nm of 0.1–0.2). Synchrony of cells in G1 or G2 phase was obtained by incubating temperature-sensitive mutants (*cdc10-M17* [47] or *cdc25–22* [48], respectively) at 36°C for 4 hours before they were released into the cell cycle at 25°C. UVC irradiation (254 nm) was performed as described previously [49]. *Hpz1:HA* and *hpz1:GFP*, were constructed using the PCR-mediated gene targeting method for fission yeast [50]. Flow cytometry was performed as described previously [31], [51] using Sytox Green to stain DNA. Aniline Blue and DAPI (4',6-diamidino-2-phenylindole) were used to stain the septa and nuclei of cells, respectively [46], [52].

### Cell Survival Assays

The spot test for survival after UVC irradiation was performed by spotting 5 µl of threefold serially diluted cultures (starting OD_595_ = 0.5) on yeast extract agar (YEA) plates. The plates were either untreated or irradiated with UVC doses of 50J/m^2^ or 300J/m^2^. A checkpoint defective mutant (*rad26*Δ) was included as a UVC-sensitive control strain.

Cell survival assays after UVC irradiation in different cell-cycle phases were performed as described previously [33]. For irradiation in G2 phase, cells were irradiated 2 hours after release from a *cdc10* block.

The cell survival assay after HU treatment was performed by incubating exponentially growing cells in 15 mM HU for 4 hours before plating onto YEA plates. Untreated cells were plated as a control.

### Immunoprecipitation

Cells were harvested by centrifugation (3000 *g*) for 5 min at 4°C and washed with STOP buffer (1× PBS, 50 mM NaF, 1 mM NaN3). The pellet was frozen in liquid nitrogen. Total cell extracts were made by adding 250µl glass beads and 200µl cold immunoprecipitation buffer (IPB) (25 mM Tris pH 7.5, 0.1 M NaCl, 10% glycerol, 0.5% NP40, 15 mM MgCl_2_, 15 mM EDTA, 60 mM glycerophosphate, 15 mM ρ-nitrophenylphosphate, 0.5 mM DTT, 1× Complete Protease Inhibitor (Roche), 1 mM Na-orthovanadate, 0.1 mM NaF) before the cells were broken using a Fast Prep (FP120, Bio 101, Thermo Electron Cooperation) for 7×20 sec at a setting of 6.5. After breakage, additional IPB (600µl) was added, cell debris pelleted and the extract cleaned by an additional centrifugation step for 15 minutes at 4°C. For immunoprecipitation 1.5 mg protein from the supernatant fraction was used. Hpz1-HA was immunoprecipitated from total cell extract with MAb 16B12 α-HA (mouse HA.11, Covance) bound to Protein G-coated Dynabeads (Dynal).

### Immunoblots

Total cell extracts for immunoblots were made by TCA protein extraction [53]. Antibodies used in this study were α-HA (1∶1000, Covance Mab 16B12), α-PSTAIRE, recognizing a motif in Cdc2 (1∶2000, Santa Cruz Biotechnology sc-53), α-c-myc (1∶1000, BD Pharmingen). Appropriate ECL kits from Amersham Biosciences were used for detection.

## Supporting Information

Figure S1 **Survival of** ***hpz1*** **Δ cells after UVC irradiation.** Survival (with standard errors from three experiments shown), of wild-type or *hpz1*Δ cells, after UVC-irradiation in G1, S or G2 phase.
(TIF)

Figure S2 **Survival of** ***hpz1*** **Δ cells after ionizing radiation.** Threefold serially diluted cultures of the indicated strains were spotted on yeast extract agar (YEA) plates. The plates were either untreated or irradiated with 300 Gy. A checkpoint defective mutant (*rad26*Δ) was included as a radiation-sensitive control strain.
(JPG)

Figure S3 **Co-immunoprecipitation of Rad3 with Hpz1.** Immunoblot showing Rad3-myc co-immunoprecipitated with Hpz1-HA. A total cell extract from G1-synchronized cells was used as a positive control for Rad3-myc presence (+). Beads without antibody was incubated with a cell extract from G1 cells to serve as a control for exclude Rad3-myc binding to the beads only (BO).
(TIF)

Figure S4 **The cell cycle of wild-type and** ***hpz1*** **Δ cells.** Percentage of wild-type or *hpz1*Δ in the different cell-cycle phases S, G2, or M-G1 in an exponentially growing culture.
(TIF)

Figure S5 **PCB boxes in the promoter region of** ***hpz1.*** A schematic display of the localization of putative PCB boxes (green) in the promoter region of *hpz1* relative to its transcription start point and the open reading frame.
(TIF)

Table S1 **Strains used in this study.**
(DOCX)
