# Supplementary figures and images for: Hpz1 Modulates the G1-S Transition in Fission Yeast
Source: PLoS One. 2012 Sep 6;7(9):e44539. doi: 10.1371/journal.pone.0044539 (PMC3435320; doi:10.1371/journal.pone.0044539)

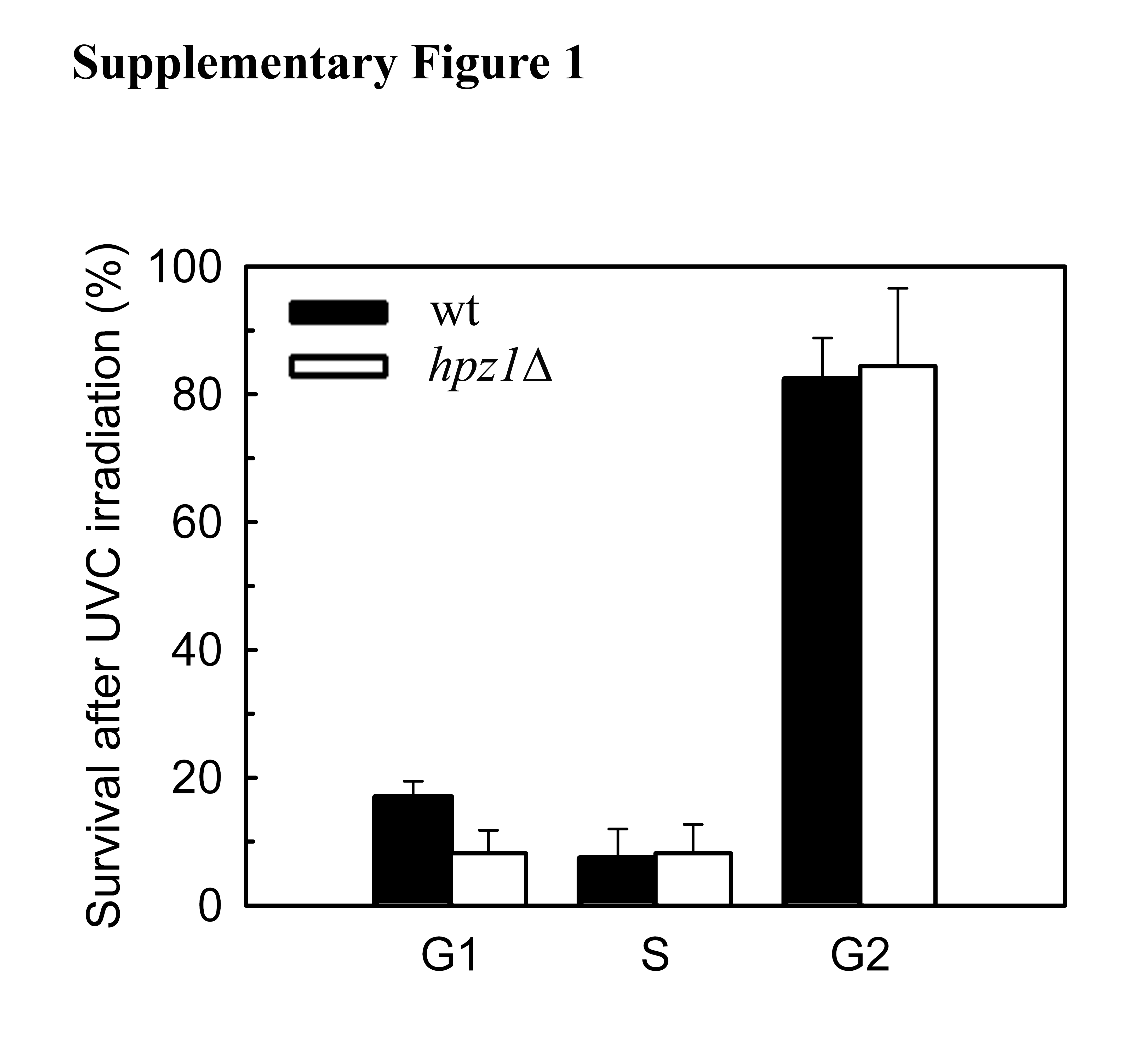

Supplement: Figure S1 — Survival of hpz1 Δ cells after UVC irradiation. Survival (with standard errors from three experiments shown), of wild-type or hpz1Δ cells, after UVC-irradiation in G1, S or G2 phase. (TIF) [file pone.0044539.s001.tif]

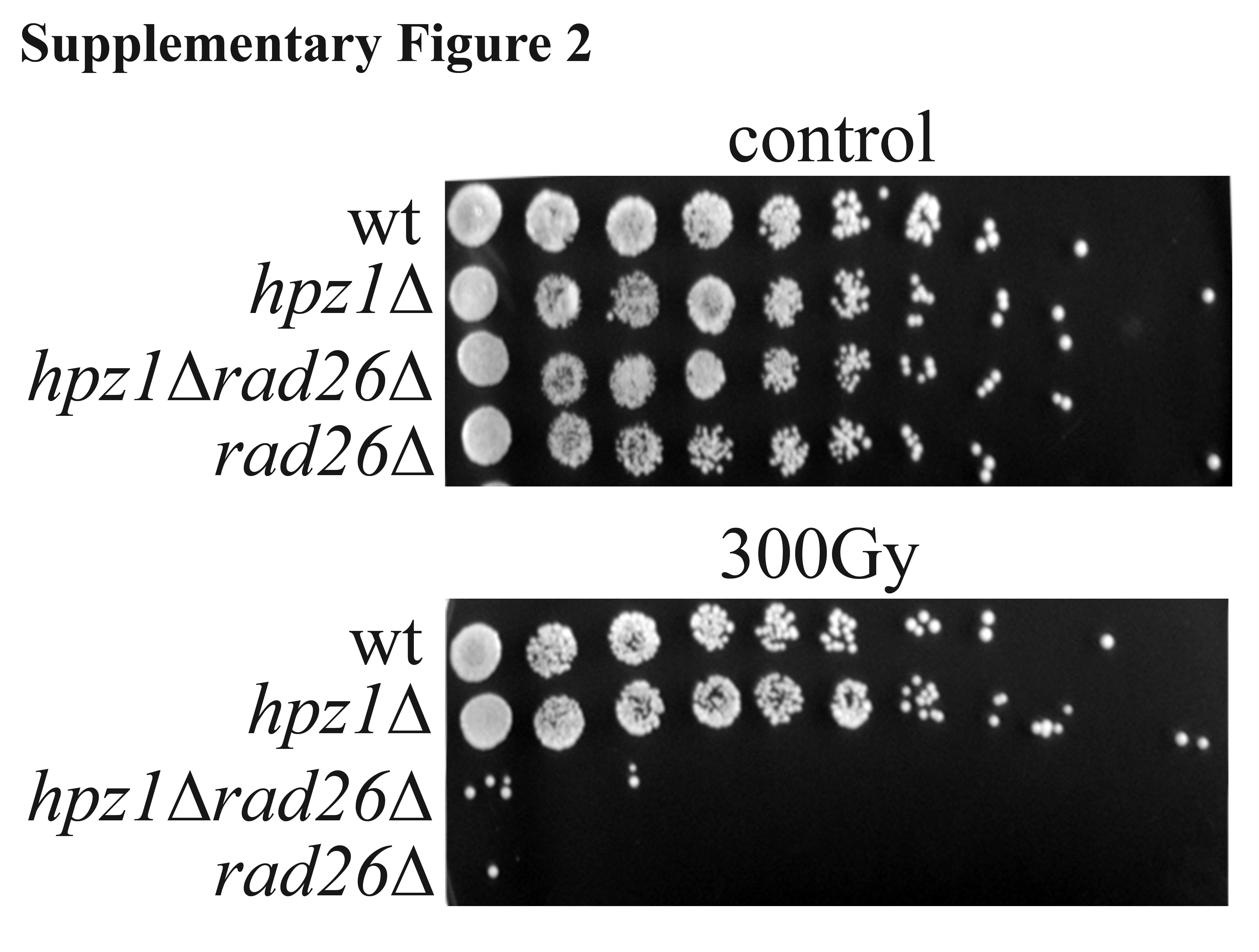

Supplement: Figure S2 — Survival of hpz1 Δ cells after ionizing radiation. Threefold serially diluted cultures of the indicated strains were spotted on yeast extract agar (YEA) plates. The plates were either untreated or irradiated with 300 Gy. A checkpoint defective mutant (rad26Δ) was included as a radiation-sensitive control strain. (JPG) [file pone.0044539.s002.jpg]

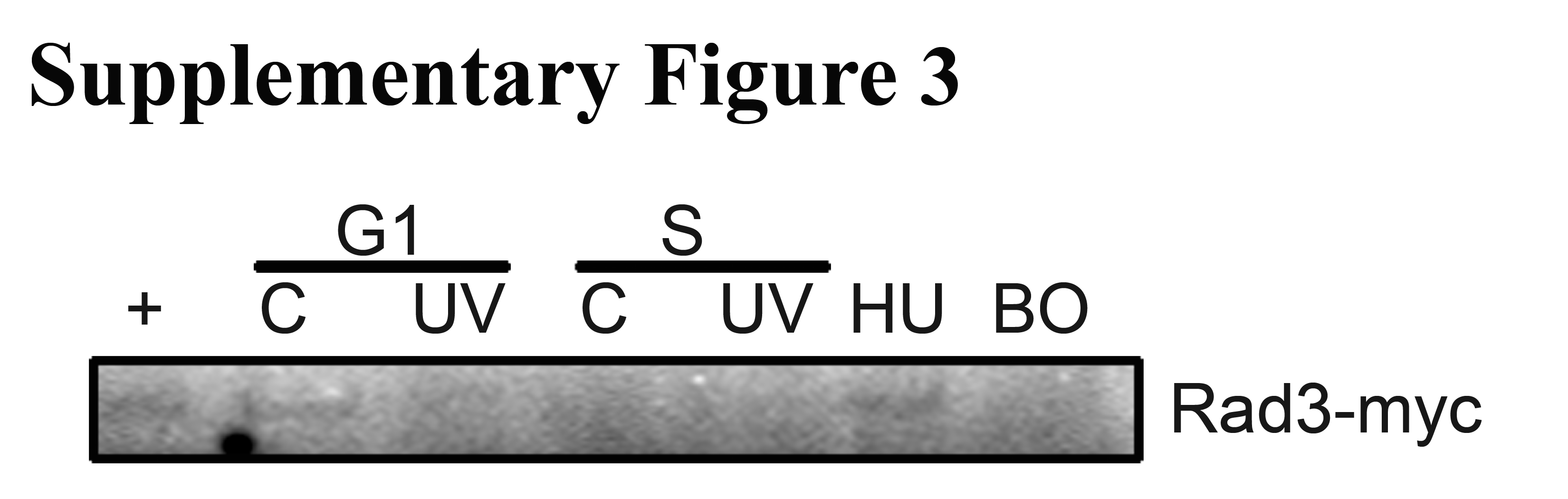

Supplement: Figure S3 — Co-immunoprecipitation of Rad3 with Hpz1. Immunoblot showing Rad3-myc co-immunoprecipitated with Hpz1-HA. A total cell extract from G1-synchronized cells was used as a positive control for Rad3-myc presence (+). Beads without antibody was incubated with a cell extract from G1 cells to serve as a control for exclude Rad3-myc binding to the beads only (BO). (TIF) [file pone.0044539.s003.tif]

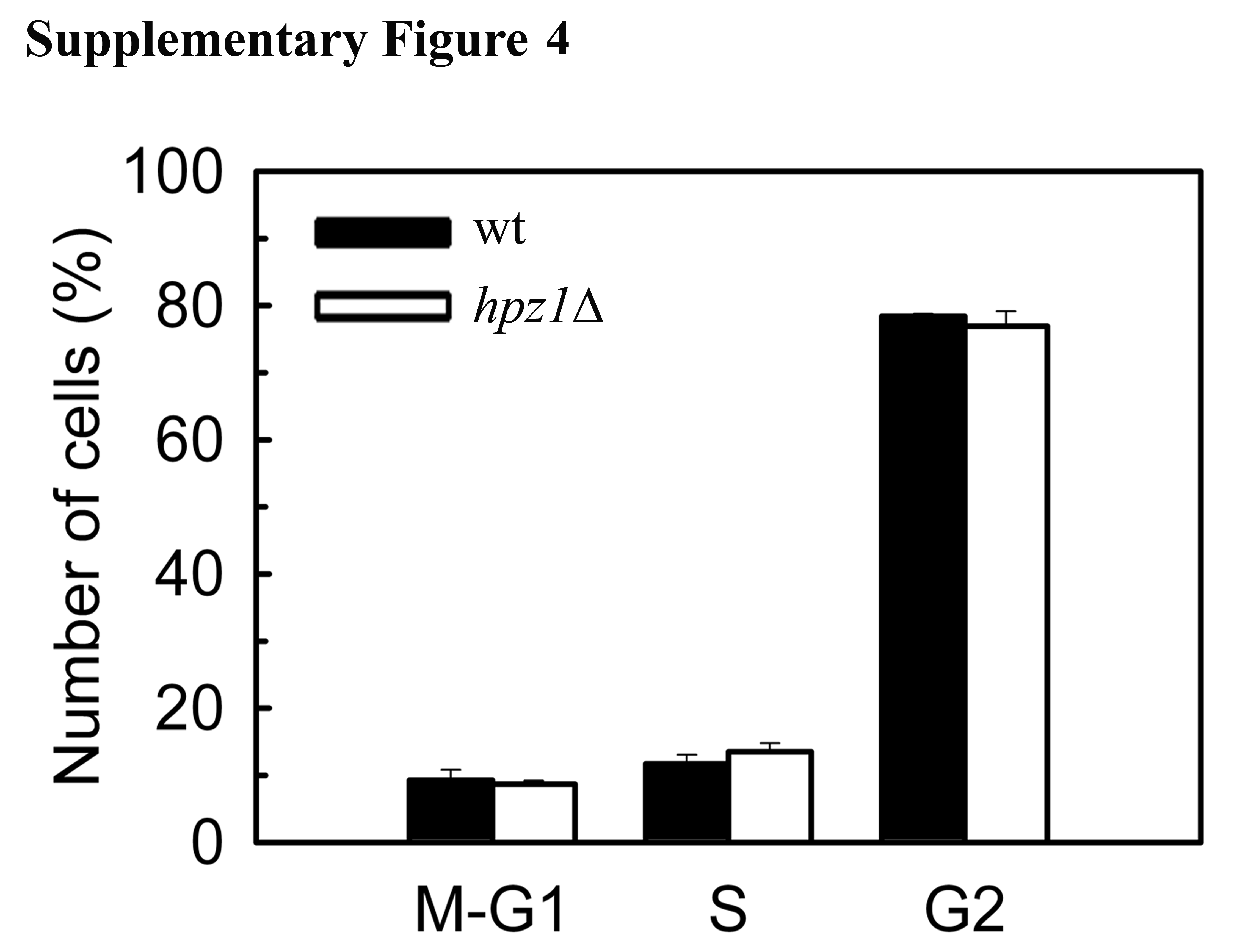

Supplement: Figure S4 — The cell cycle of wild-type and hpz1 Δ cells. Percentage of wild-type or hpz1Δ in the different cell-cycle phases S, G2, or M-G1 in an exponentially growing culture. (TIF) [file pone.0044539.s004.tif]

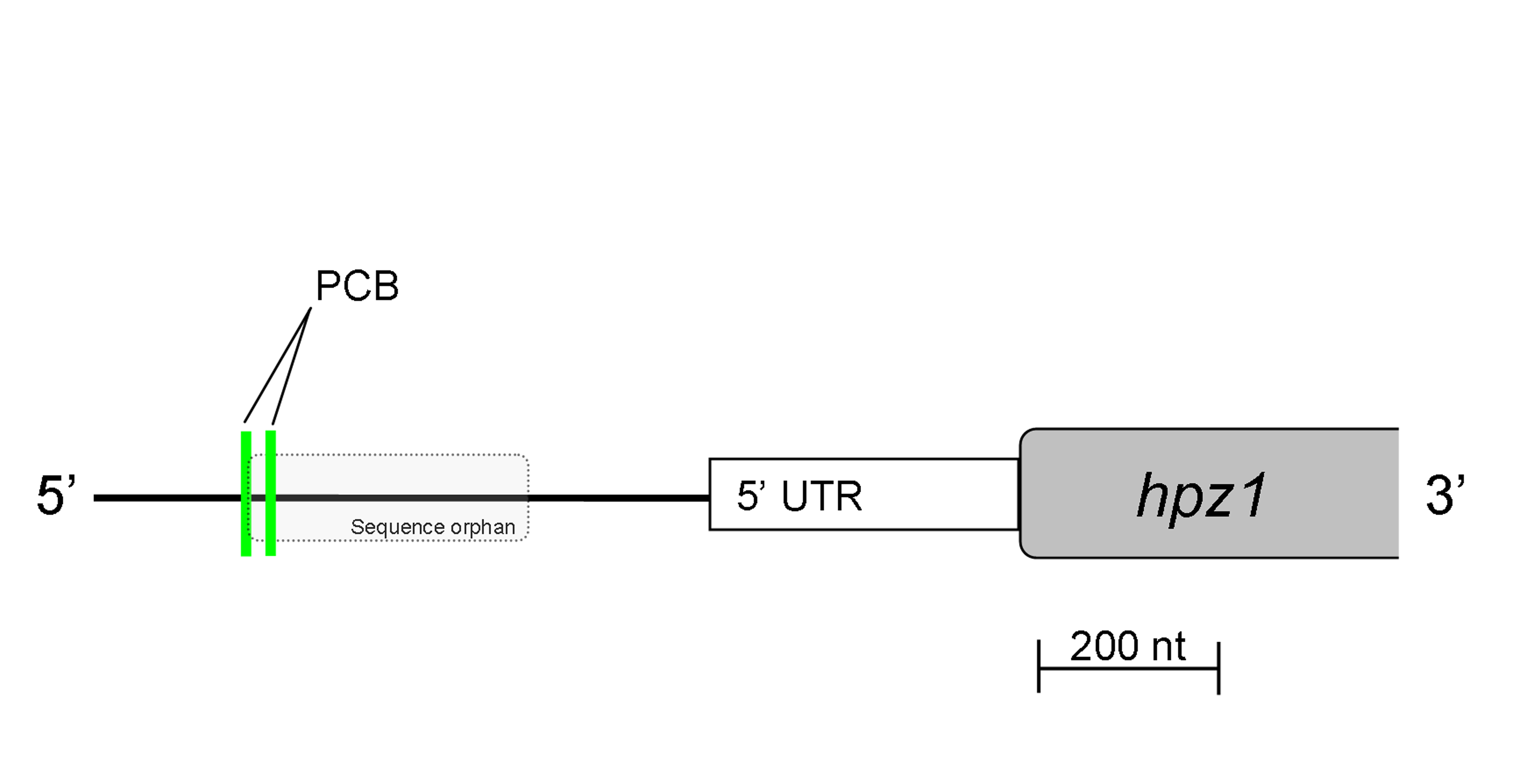

Supplement: Figure S5 — PCB boxes in the promoter region of hpz1. A schematic display of the localization of putative PCB boxes (green) in the promoter region of hpz1 relative to its transcription start point and the open reading frame. (TIF) [file pone.0044539.s005.tif]
