# Supplementary material for: Hpz1 Modulates the G1-S Transition in Fission Yeast
Source: PLoS One. 2012 Sep 6;7(9):e44539. doi: 10.1371/journal.pone.0044539 (PMC3435320; doi:10.1371/journal.pone.0044539)
Supplement: Table S1 — Strains used in this study. (DOCX) [file pone.0044539.s006.docx]

**Supporting Table S1. Strains used in this study**

| **Strain** | **Genotype** | **Source** |
| --- | --- | --- |
| 489 | cdc10-M17 | P. Nurse |
| 1340 | hpz1::kanMX4 ura4-D18 h- | Bioneer [54] |
| 1337 | cdc10-M17 hpz1::kanMX4 ura4-D18 h+ | This work |
| 996 | rad26::ura4+ ade1-D25 ura4-D18 h+ | Lab collection |
| 1417 | cdc10-M17 rad3:myc hpz1:3HA:kanR ade6-704 ura4-D18 | This work |
| 983 | cdc10-M17 cdc21:GFP h- | Lab collection |
| 1448 | hpz1::kanMX4 cdc10-M17 cdc21:GFP:ura4+ ura4-D18 | This work |
| 1375 | hpz1:GFP:clonnatMX6 cdc10-M17 h- | This work |
| 1418 | cdc25-22 hpz1:HA:kanR | This work |
| 1379 | cdc10-M17 hpz1:3HA:kanMX6 h- | This work |
